# Leishmanicidal and immunomodulatory activities of the formononetin (a natural isoflavone) against *Leishmania tropica*

**DOI:** 10.1186/s13104-023-06403-1

**Published:** 2023-06-26

**Authors:** Hossein Mahmoudvand, Amal Khudair Khalaf, Pouya Zareh Rajabi, Nooshin Karbasian, Javad Ghasemian Yadegari

**Affiliations:** 1 Razi Herbal Medicines Research Center, Lorestan University of Medical Sciences, Khorramabad, Iran; 2 Department of Microbiology, College of Medicine, University of Thiqar, Thiqar, Iraq; 3 Student Research Committee, Lorestan University of Medical Sciences, Khorramabad, Iran

**Keywords:** Leishmaniasis, Promastigote, Amastigote, Nitric oxide, Infectivity

## Abstract

**Objective:**

This work aimed to examine the leishmanicidal, cellular mechanisms and cytotoxicity effects of formononetin (FMN), a natural isoflavone, against *Leishmania tropica*. We used the MTT assay to determine the leishmanicidal effects of FMN against promastigotes and its cytotoxicity effects on J774-A1 macrophage cells. The Griess reaction assay and quantitative real-time PCR were used to determine the nitric oxide (NO) and the mRNA expression levels of IFN-γ and iNOS in infected J774-A1 macrophage cells.

**Results:**

FMN significantly (P < 0.001) decreased the viability and number of promastigotes and amastigotes forms. The 50% inhibitory concentrations value for FMN and glucantime was 9.3 and 14.3 µM for promastigote and amastigote, respectively. We found that the macrophages exposed with FMN especially at concentrations of 1/2 IC_50_ and IC_50_ significantly activated the NO release and the mRNA expression levels of IFN-γ, iNOS. The findings of the current research showed the favorable antileishmanial effects formononetin, a natural isoflavone, against various stages of *L. tropica* through inhibition of infectivity rate of macrophage cells and triggering the NO production and cellular immunity. However, supplementary works are essential to evaluate the ability and safety of FMN in animal model before use in the clinical phase.

## Introduction

Leishmaniasis is a parasitic infection which found in various parts of the worlds as a neglected tropical disease (NTD). The disease is caused by *Leishmania* parasites, which are transmitted by the bite of phlebotomine sand flies [1]. Leishmaniasis can be clinically separated into four categories: cutaneous, muco-cutaneous, diffuse and kala-azar [2]. The cutaneous form as more frequent form is found in some countries such as Iran, Saudi Arabia, Syria, Iraq [3]. Because, there is no effective vaccine to prevent this disease, treatment of patients especially using synthetic drugs, is one of the most important ways to break the chain of transmission and prevent the disease [4, 5]. The most synthetic drugs available are the use of pentavalent antimony compounds [5]. Glucantime (meglumine antimoniate, MA) is the most common drug; whereas the use of this drug is linked with common complications such as anorexia, fever, chills, and joint pain usually in patients with liver and kidney problems [6]. In addition, the high cost and drug resistance to this drug results in the efforts for discovering the alternative therapies [7]. Todays, it has been proven that natural products are a main rich source of anti-infective agents [8]. Flavonoids as the main polyphenolic compounds are widely present in the herbs with a wide range of pharmacological and therapeutic properties [9]. Isoflavones with the chemical formula (C_20_H_18_O_6_) are a subset of flavonoid compounds with various beneficial effects on human health and in particular prevention of cancer and cardiovascular disease and reduction of the symptoms of menopause [10]. Formononetin (7-Hydroxy-4’-methoxy-isoflavone, C_16_H_12_O_4_) is a natural isoflavone found in low concentrations in many foods belonging to the Fabaceae family [11]. Formonontin (FMN) exhibited some properties in modern medicine, e.g., antimicrobial, antioxidant, anti-hyperlipidemic, anti-hyperlipidemic, anti-diabetic, anti-tumor, neuroprotective, and cardioprotective activity [12, 13]. With respect to various biological effects of FMN, this work intended to examine the in vitro anti-leishmanial effects and cytotoxicity effects of FMN on *Leishmania tropica*.

## Materials and methods

### Chemicals

Formononetin (purity > 99%), Griess reagent, Fetal Bovine Serum (FBS), 1640 RPMI medium, Eosin powder were prepared from Sigma-Aldrich, Germany. Giemsa powder and dimethyl sulfoxide (DMSO) were also prepared from Merck, Germany.

### Cell and parasite

*L. tropica* (MHOM/AF/88/KK27) and macrophage cells (J774-A1 cell lines, Pasteur Institute, Iran) were cultured in 1640 RPMI medium with FBS (10%), penicillin/streptomycin (100 mL/IU) at 24 ± 1 and 37 °C, respectively.

### Effect of FMN on promastigotes

Initially, promastigotes (1 × 10^6^/cells) were treated with FMN (6.25–100 µg/mL) at 24 ° C for 48 h. After discarding the supernatant of the mixture in tested wells, 20 µL of MTT solution (0.5 mg/mL) was added and wells kept again 5% CO_2_ at 37 °C for 4 h. followed by adding DMSO (50 µL) the absorbance of solution was read at 570 nm by an ELISA plate reader [14]. Amphotericin B (AmB) and non-treated parasites were considered as the positive and control groups. The half maximal inhibitory concentration (IC_50_) values were calculated by Probit test in SPSS software ver. 26.0.

### Effect of FMN on amastigotes

One hundred µL of promastigotes (1 × 10^6^/mL) in stationary phase (10:1) were exposed with macrophage cells (1 × 10^5^/mL) at 37 °C in 5% CO_2_ for 24 h. Then, macrophages were exposed with FMN (6.25–200 µM) and MA for 48 h. After preparing the slides and staining by Giemsa dye, the number of amastigotes were recorded [15].

### Cytotoxic effects of FMN on macrophages

Cytotoxic effects of FMN on normal macrophage cells was performed similar to the effect of FMN on promastigotes based on the MTT assay. The selectivity index (SI) was recorded through the CC_50_ /IC_50_ for amastigotes [16].

### Effect of FMN on the infectivity rate

Briefly, *L. tropia* promastigotes (1 × 10^6^/mL) were pre-treated with FMN at ¼ (2.35 µM), 1/3 (3.1 µM), and ½ IC_50_ (4.65 µM) for 120 min at 21 °C. Then, parasites were exposed to macrophages cells for one day. After preparing the slides and staining by Giemsa dye, the number of infected macrophages were recorded [16].

### Effect on nitric oxide (NO) generation

The macrophage cells (1 × 10^5^/mL) were incubated with FMN at ¼ (3.5 µM), 1/3 (4.7 µM), and ½ IC_50_ (7.15 µM) for two days, then, the collected supernatants (100 µL) along with 50 µL of the Griess reagent A and B were added to the tested wells. The absorbance of microplates was recorded by an ELISA reader at 540 nm [17]. lipopolysaccharide (10 ng/ml) + IFN-γ (10 U/ml) were reflected as the positive control.

### Genes expression of (iNOS and IFNγ) in infected macrophages

The mRNA expression levels of IFN-γ and iNOS in infected J774-A1 macrophage cells treated with FMN at ¼ (3.5 µM), 1/3 (4.7 µM), ½ IC_50_ (7.15 µM) and IC_50_ (14.3 µM) were examined by quantitative real-time PCR. At first total RNA was extracted based on the instructions of RNeasy tissue kit (Qiagen, Germany). Next, the complementary DNA (cDNA) was obtained through random primers according to the instructions of the commercial kit (Qiagen, Germany). Lastly, the obtained cDNA was considered for real-time PCR through SYBR green. The thermal cycle for this experiment was 92 °C for 6 min, 42 cycles of 92 °C for 10 s and 55 °C for 30 s, respectively. The iQTM5 optical system software (Bio-Rad, Hercules, CA) was applied for the ΔCt^− 2^ and the results were standardized by the value found from the β-actin mRNA. The oligonucleotide primers for real-time PCR was prepared based on the study conducted by Gharavi et al. [18].

### Statistical analysis

To increase the validity of the results all tests were repeated in triplicate. The findings were analyzed by SPSS software (version 26.0). One-way analysis of variance (ANOVA) was also performed to data analysis. P < 0.05 revealed the significance level. All examinations were carried out for three times.

## Results

**Fig. 1:**
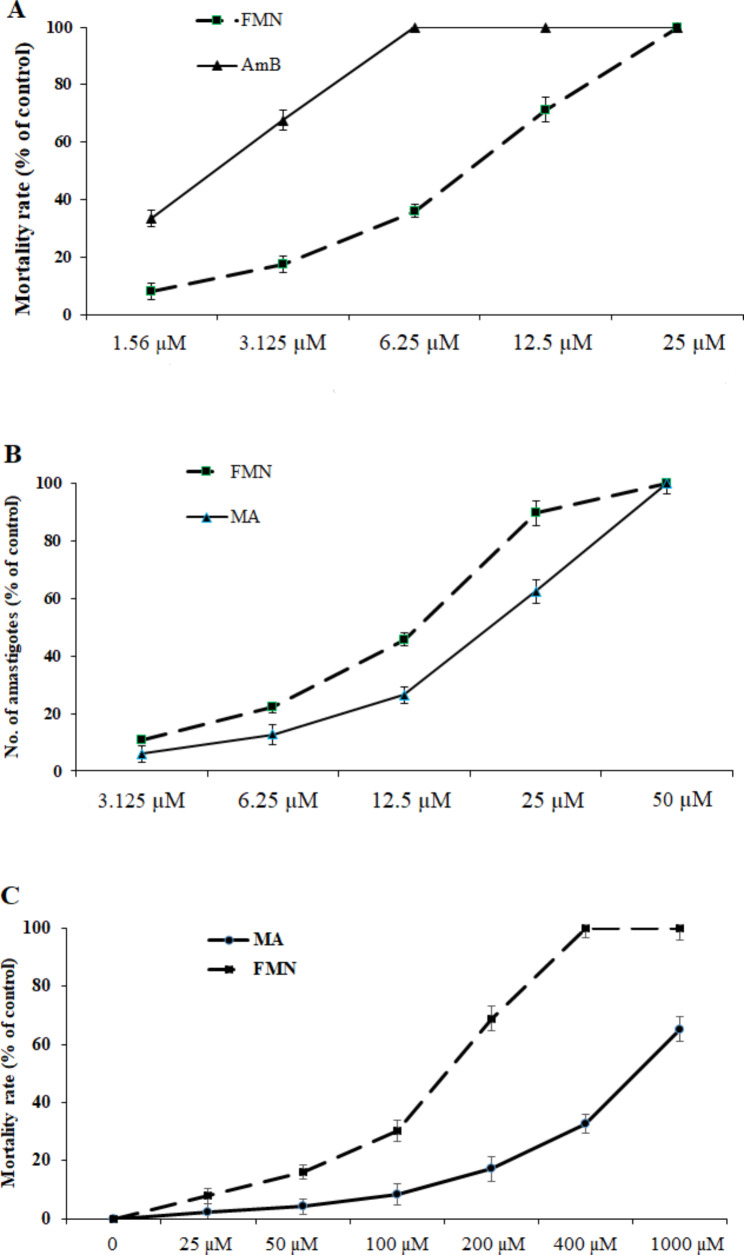
Inhibitory effects of formononetin (FMN), meglumin antimoniate (MA), and amphotericin B (AmB) against *Leishmania tropica* promastigote **(A)**, amastigote **(B)**, and macrophage cells **(C)**. Mean ± standard deviation. (n = 3)

**Fig. 2:**
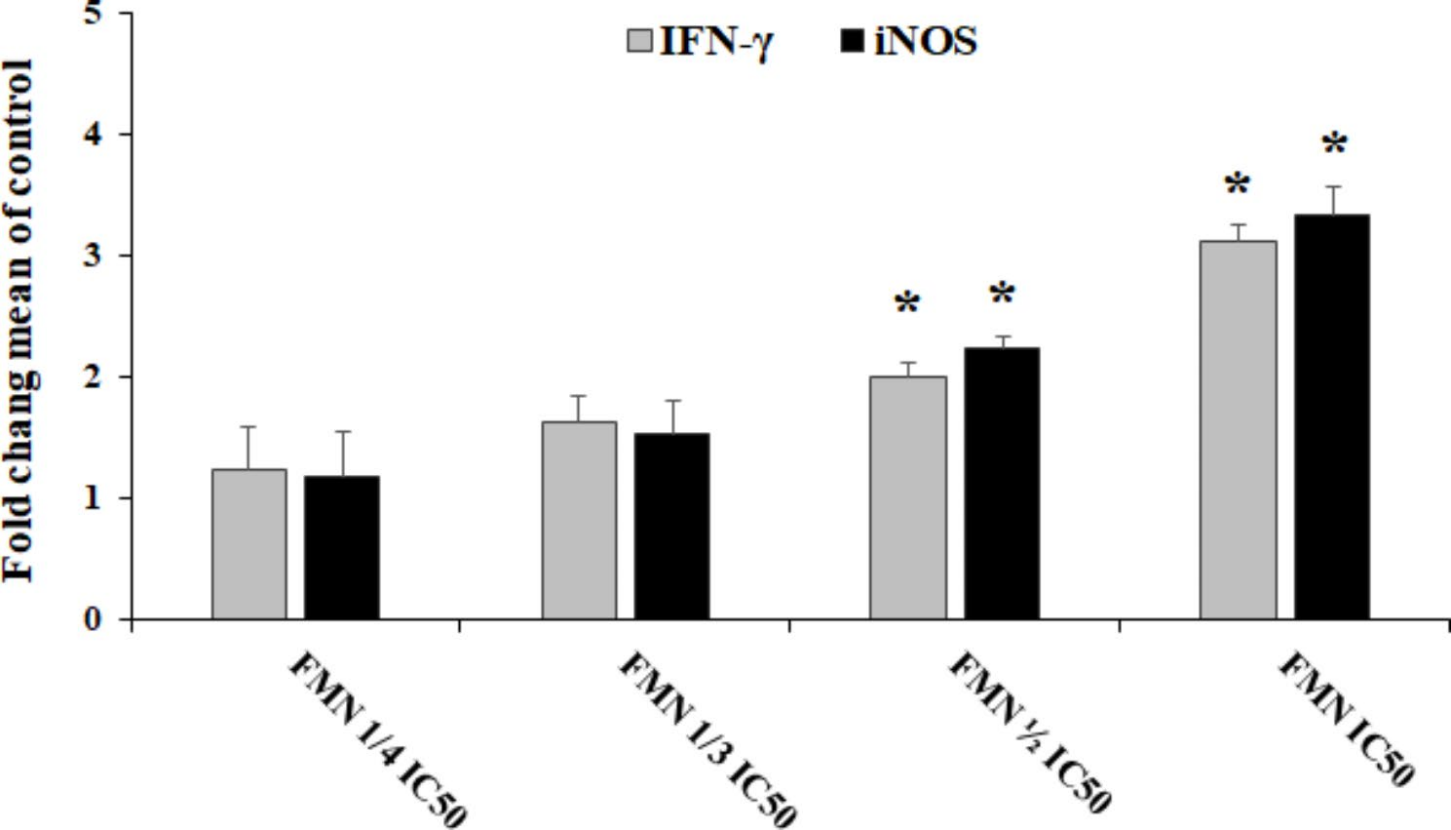
mRNA expression of genes (iNOS and IFNγ) in infected macrophages with *Leishmania tropica* promastigotes treated with formononetin (FMN) in comparison with the control group. mean ± SD (n = 3). * p < 0.001

Based on the findings of the MTT assay (Fig. 1A), the FMN significantly (P < 0.001) reduced the viability of *L. tropica* promastigotes compared with the negative control. Furthermore, the IC_50_ levels for FMN and AmB were 9.3 µM and 2.31 µM, respectively. According to the results of the macrophage model (Fig. 1B), FMN represented the considerable leishmanicidal activity on amastigote forms as dose-dependent response. FMN markedly inhibited the mean number of intracellular amastigotes into macrophage cells as a dose dependent response in comparison to the non-treated control group (p < 0.001). The IC_50_ values for the FMN and MA were 14.3 and 26.2 µM, respectively. The cytotoxicity of various concentrations of the FMN and MA on mice macrophage cells was evaluated by MTT assay (Fig. 1C). The CC_50_ level of the FMN and MA was 159.3 and 874.6 µM, respectively. The findings of microscopic assay displayed (Table 1) that percent of infected macrophages by no pre-treated promastigotes was 81.6 ± 3.7; while, after pre-treatment of promastigotes with the FMN at ¼ IC_50_, 1/3 IC_50_, 1/2 IC_50_ indicated the rate of cell infection was declined by 8.9, 34.5, and 64.9%, respectively (p < 0.05). As displayed in Table 1, the macrophages exposed with FMN activated the NO release, but a significant (p < 0.001) rise was reported at 1/3 IC_50_ and ½ IC_50_ compared to the control group. As shown in Fig. 2, the mRNA expression levels of IFN-γ and iNOS in infected J774-A1 macrophage cells treated with AMCE especially at ½ IC_50_ and IC_50_ was significantly (p < 0.001) elevated in quantitative real-time PCR.

**Table 1:** The effect of formononetin (FMN) on nitric oxide (NO) generation and infectivity rate in J774-A1 macrophages cells in comparison with the control groups. IFN-γ: gamma interferon; LPS: Lipopolysaccharide; MA: meglumine antimoniate. Mean ± SD

Material	NO production (nM)	Infectivity rate		
		% of infected macrophages	% of reduction	
¼ IC_50_	3.11 ± 0.47	74.3 ± 4.16	8.9	
1/3 IC_50_	4.24 ± 0.85*	53.4 ± 3.88	34.5	
1/2 IC_50_	12.3 ± 1.42*	28.6 ± 2.51	64.9*	
Non-treated	2.49 ± 0.31	81.6 ± 1.42	-	
IFN-γ + LPS	30.24 ± 4.45	-		-
MA	-	32.3 ± 3.21		-

* *p* < 0.001

## Discussion

Based on our findings, the FMN significantly (P < 0.001) declined number and viability of promastigotes and amastigotes forms of *L. tropica* compared with the negative control. In line with our results, previous study showed the effective antimicrobial activity of FMN against some pathogenic bacterial (*Staphylococcus aureus, S. aureus, S. epidermides*, and *Pseudomonas aeruginosa*) and fungal (*Candida albicans, C. tropicalis, Cryptococcus neoformans*) strains with values ranging from 25 to 200 µg/mL [19]. Yang Ying et al. (2008), showed the antibacterial effects of FMN against the gram-positive and the gram-negative bacteria [20]. Wang et al. (2015) indicated the antiviral effects of FMN on enterovirus-51, they fund that FMN fraction can dose-dependently reduce enterovirus-51 RNA and protein synthesis [21]. Lauwaet et al. (2010) revealed that FMN significantly inhibited not only attachment and flagellar motility but also the number of *Giardia* trophozoites in mice after 1.5 h treatment [22]. Mead and McNair (2006) have showed that among some tested flavonoids and isoflavones compounds (e.g., galangin, galangin, luteolin, sylibinin, quercetin, genistein, apigenin, and narigenin), quercetin, apigenin, and genistein revealed the highest in vitro antiparasitic effects against *Cryptosporidium parvum* and *Encephalitozoon intestinalis* with effective concentration (EC_50_) values ranging from 5.5 to 15 µM [23]. Recently, Faixová et al. (2021) have reported the antiparasitic effects of a number of Isoflavones (genistein, curcumin, silymarin, and quercetin) against some flatworms, e.g., *Raillietina* spp., *Fasciola* spp., *Schistosoma* spp., and *Echinococcus* spp through affecting the tegumental construction and disturbance in metabolism by interaction with enzymes or signaling molecules [24]. Although, the antimicrobial mechanism of action of isoflavonoids, such as formonontin, has not yet been reported; but, previous studies reported that isoflavones act mainly through the disrupting the cell permeability which subsequently caused the leakage of vital metabolites minerals, and contents, e.g., amino acids, ions, calcium [9–12, 25]. Kaur et al. (2021) showed the promising *in vitro* and *in vivo* effects of *Bauhinia variegate*, a flavonoid-rich plant, against *L. donovani* through cell cycle arrest at sub-G0/G1 phase, the improvement of disease-suppressing Th1 cytokines and inhibition of disease-progressing Th2 cytokines with no toxicities [26]. It has been proven that macrophage cells are the key immune cells to control and removing *Leishmania* parasites. Macrophage cells by the triggering NO synthesis and then release of NO lead to elimination and controlling of the *Leishmania* parasite [27]. NO is one of the most important effector molecules that rise due to IFN-gamma stimulation or intracellular infection in macrophages along with reactive oxygen species (ROS) production [27, 28]. At the level of cell signaling, SHP-1 phosphatases have been shown to have major biological role in controlling *Leishmania* in macrophages [29]. On the other hand, inhibition of contamination in macrophages cells is well-known as vital mechanisms targeted by means of drugs for controlling of *Leishmania* [18]. We found that the macrophages exposed with FMN especially at concentrations of 1/3 IC_50_ and ½ IC_50_ significantly activated the NO release. In addition, we reported that the mRNA expression levels of IFN-γ and iNOS in infected J774-A1 macrophage cells treated with AMCE especially at ½ IC_50_ and IC_50_ was significantly (p < 0.001) elevated in quantitative real-time PCR Our findings also displayed that pre-treatment of promastigotes with FMN markedly dropped the rate of infectivity by 60.3%. Considering the cytotoxicity effects of FMN, the cytotoxicity of various concentrations of the FMN and MA on mice macrophage cells was evaluated by MTT assay. The CC_50_ level of the FMN and MA was 159.3 and 874.6 µM, respectively. Consequently, the obtained SI of > 10 for FMN and MA represented their specificity to *L. tropica* amastigotes with lowest harmfulness on macrophage cells.

## Conclusion

The findings of the current research showed the promising in vitro antileishmanial effects formononetin, a natural isoflavone, against promastigote and amastigote forms of *L. tropica* through inhibition of infectivity rate of macrophage cells and triggering the NO production. However, supplementary works are essential to evaluate the ability and safety of FMN in animal model before use in the clinical phase.

### Limitation

The main limitations of this study are the lake of in vivo study and others cellular mechanis of action of FMN.

## Data Availability

All data generated or analysed during this study are included in this published article.

## Abbreviations

NO: nitric oxide
ANOVA: One-way analysis of variance (ANOVA)
MTT: 3-(4,5-Dimethylthiazol-2-yl
MA: meglumine antimoniate
DMSO: dimethyl sulfoxide
SI: the selectivity index
IC_50_: The half maximal inhibitory concentration
